# Mechanical Ventilation Alters the Development of *Staphylococcus aureus* Pneumonia in Rabbit

**DOI:** 10.1371/journal.pone.0158799

**Published:** 2016-07-08

**Authors:** Saber-Davide Barbar, Laure-Anne Pauchard, Rémi Bruyère, Caroline Bruillard, Davy Hayez, Delphine Croisier, Jérôme Pugin, Pierre-Emmanuel Charles

**Affiliations:** 1 Laboratoire “Ventilation Immunité Poumon”, Pôle Microbiologie Environnementale et Risque Sanitaire (M.E.R.S.), U.M.R. 1347, I.N.R.A., Université de Bourgogne, Dijon, France; 2 Vivexia S.A.R.L., Gemeaux, France; 3 Intensive Care Laboratory, University Hospitals of Geneva, and Department of Microbiology and Molecular Medicine, Faculty of Medicine, 1211 Geneva 14, Switzerland; University of Bari, ITALY

## Abstract

Ventilator-associated pneumonia (VAP) is common during mechanical ventilation (MV). Beside obvious deleterious effects on muco-ciliary clearance, MV could adversely shift the host immune response towards a pro-inflammatory pattern through toll-like receptor (TLRs) up-regulation. We tested this hypothesis in a rabbit model of *Staphylococcus aureus* VAP. Pneumonia was caused by airway challenge with *S*. *aureus*, in either spontaneously breathing (SB) or MV rabbits (n = 13 and 17, respectively). Pneumonia assessment regarding pulmonary and systemic bacterial burden, as well as inflammatory response was done 8 and 24 hours after *S*. *aureus* challenge. In addition, *ex vivo* stimulations of whole blood taken from SB or MV rabbits (n = 7 and 5, respectively) with TLR2 agonist or heat-killed *S*. *aureus* were performed. Data were expressed as mean±standard deviation. After 8 hours of infection, lung injury was more severe in MV animals (1.40±0.33 *versus [vs]* 2.40±0.55, *p* = 0.007), along with greater bacterial concentrations (6.13±0.63 vs. 4.96±1.31 colony forming units/gram, *p* = 0.002). Interleukin (IL)-8 and tumor necrosis factor (TNF)-αserum concentrations reached higher levels in MV animals (*p* = 0.010). Whole blood obtained from MV animals released larger amounts of cytokines if stimulated with TLR2 agonist or heat-killed *S*. *aureus* (e.g., TNF-α: 1656±166 vs. 1005±89; *p* = 0.014). Moreover, MV induced TLR2 overexpression in both lung and spleen tissue. MV hastened tissue injury, impaired lung bacterial clearance, and promoted a systemic inflammatory response, maybe through TLR2 overexpression.

## Introduction

Mechanical ventilation (MV) is sometimes the only way to care for critically ill patients with respiratory failure, many adverse effects, including ventilator-associated pneumonia (VAP), are common [1, 2]. Actually, VAP occurs in up to 27% of these patients (23.5 per 1,000 ventilator-days) [3]. During bacterial pneumonia, a rapid immune lung response is necessary to ensure microbial clearance. In the airways, both bronchial and alveolar epithelial cells take part in the host innate immune response, mainly through their ability to produce inflammatory mediators [4]. It is now clearly recognized that innate immunity is activated through pattern recognition receptor-related pathways [5]. Among the pattern recognition receptors, toll-like receptors (TLRs) play a key role in the recognition of the so-called pathogen-associated microbial patterns. TLRs can recognize a wide range of pathogen-associated microbial patterns and generate inflammatory signals to modulate innate immune responses [6]. Among the bacteria causing VAP, Gram-positive bacteria, especially *Staphylococcus aureus*, are frequently responsible for infection and mortality [7]. Cell wall components of Gram-positive bacteria, such as bacterial lipopeptides, activate TLR2. TLR2 expression has been detected in immune cells, as well as in endothelial or epithelial cells [8]. Ligand-specific recognition and signaling occurs via heterodimerisation with TLR1 or TLR6 [6, 9]. Interestingly, TLR2 expression seems to be sensitive to mechanical stress, as shown in the endothelium [10].

Both *in vitro* and *in vivo* experimental studies have demonstrated that MV, in particular adverse ventilatory strategies with high tidal volume and zero end-expiratory pressure could activate lung cells, thus leading to a proinflammatory response, even in the absence of bacterial stimuli. This is the biotrauma paradigm, which is responsible, at least in part, for so-called Ventilator Induced Lung Injury (VILI), the hallmark of which is the infiltration of polymorphonuclear neutrophils (PMNs) into the lungs [11]. Although low-stretch MV does not lead to such apparent tissue damage or inflammation, it could prime airway cells to respond massively to a second proinflammatory insult, through the subsequent release of large amounts of cytokines, thus leading to additional lung injury, particularly through the recruitment of neutrophils as a result of interleukin (IL)-8 secretion [12]. This has been clearly demonstrated in several animal models based on airway challenge with lipopolysaccharide (LPS) combined with MV. It has been shown that MV increased lung injury and promoted pulmonary-to-systemic endotoxin translocation [13–15]. Innate immunity activation thereby plays a major role in the development of VILI [5]. The involvement of toll-like receptors, especially TLR4, has been suggested by several experimental studies [16, 17]. Moreover, it has been hypothesized that MV-dependent lung priming depended on the TLR4 pathway when LPS models were used, thus illustrating the “two-hit” paradigm [13–15, 18]. However, little is known about the possible implication of other TLRs and related microbial ligands in MV-dependent lung injury.

Indeed, we have previously demonstrated *in vitro* that cyclic stretch of human pulmonary cells resulted in TLR2 overexpression and enhanced TLR2 reactivity to Gram-positive cell wall components [19]. These findings were confirmed in a rabbit model since MV increased lung injury in response to the instillation of a specific TLR2 agonist. Our findings suggested that MV could prime the lung, through TLR2 up-regulation, promoting thereby lung inflammation and injury in the case of subsequent infectious insult. In an attempt to determine the relevance of this finding in the pneumonia setting, as well as its potential impact on the antimicrobial host defense, we conducted experiments using live Gram-positive bacteria specifically recognized by TLR2 (i.e., *S*. *aureus*)

## Materials and Methods

### Bacterial Challenge

The methicillin-resistant *S*. *aureus* (MRSA) USA 300 Panton Valentine Leukocidin (PVL) (negative) clinical strain (kindly provided by G. Lina, Lyon, France) was used in this study. Working stock cultures were kept frozen in a 15% glycerol-supplemented brain-heart infusion (BioMérieux Laboratories, Marcy-l’Etoile, France). They were changed monthly. Colonies isolated from one infected rabbit’ spleen cultures were used to preserve bacterial virulence (as planned into the protocol submitted to the Ethical committee). Actually, *S*. *aureus* pneumonia was induced and sacrifice occurred 48 hours later, as described below. Several bacterial colonies isolated from spleen culture were then collected and inoculated into brain-heart infusion. After 6-hour incubation at 37°C, one bacteria suspension was obtained. Aliquots were then prepared and stored at -80°C.

At 48 hours before experimentation began, several colonies were taken, cultured on MRSA2 agar plates (BioMérieux) and then incubated for 24 hours at 37°C. One colony was inoculated into 10 milliliters (mL) of brain-heart infusion and was incubated for 6 hours at 37°C before being cultured on MRSA2 agar plates for 18 hours at 37°C. The inocula concentration was estimated by optical densitometry, in reference to a standard curve, and systematically checked by a quantitative culture by plating successive ten-fold dilutions. A mean titer of 9.5 log_10_ colony-forming units (CFU) per mL was used in this study since pneumonia obtained with lower values was too mild and transient [20].

### Animal experiments

Male New Zealand White rabbits (body weight 2.8 to 3.2 kilograms [kg]) were obtained from the “Elevage scientifique des Dombes” (Romans, France). They were placed in individual cages and were fed ad libitum with water and food, according to current recommendations. The Ethics Committee of Dijon Faculty of Medicine approved the experimental protocol (N. 0810). A central venous catheter was surgically inserted into the left jugular internal vein 24 hours before randomization.

**Fig 1:**
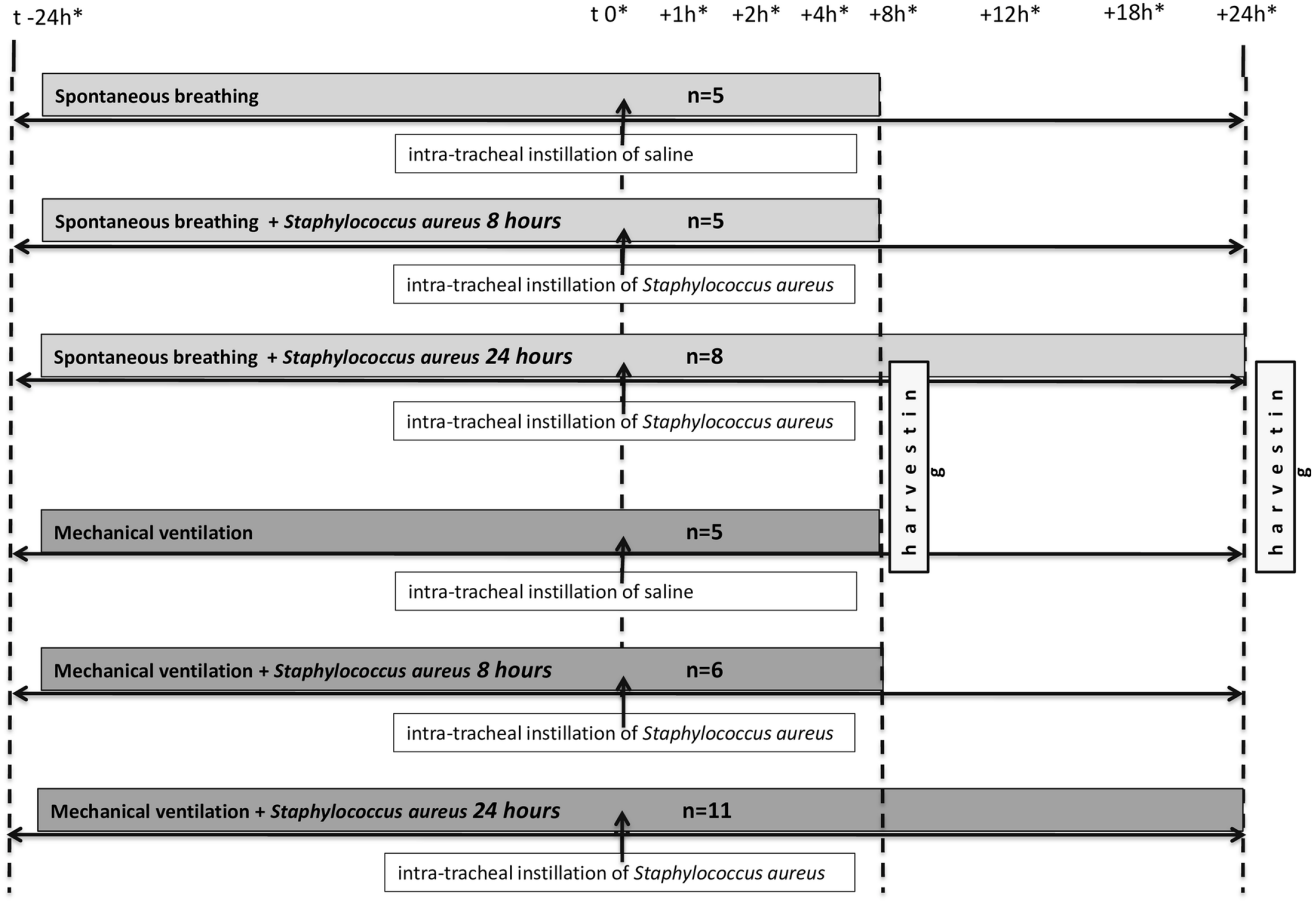
Timeline representation of the experimental protocol. *denotes blood sample drawing.

The animals (n = 40) were randomly allocated to the SB group or the MV group (n = 18 and 22, respectively) (Fig 1).

In the spontaneously breathing (SB) group, the rabbits were orally intubated as previously described [21, 22]. Briefly, under general anaesthesia provided by an intravenous injection of ketamine 3.3 milligrams (mg)/kg (Panpharma, France) and xylazine 1 mg/kg (Rompun^®^, Bayer, Germany), a cuff tube of 3.0 mm was orally inserted into the trachea under view control. Unscheduled death occurred during the procedure in 4 rabbits, accounting for the fact that there was not equal number of animals in every concurrent group. Those deaths were related to the general anaesthesia procedure itself since unexpected drug adverse effects might occur in some rabbits.

The SB rabbits then received an intrabronchial instillation of 9.5 log_10_ CFU/mL of *S*. *aureus* diluted in 0.5 mL of saline (n = 5), through a silicon catheter (Sigma Medical, Nanterre, France) inserted into the tracheal tube. The inoculum was gently flushed through the catheter until one lower lobe bronchus was reached. Spontaneously breathing controls received 0.5 mL of saline (n = 5). The catheter was immediately removed, the animals were rapidly extubated, and were then allowed to go back to their cage before being killed 8 or 24 hours later (n = 5 and 8, respectively).

In the MV group, the animals were intubated as described above, and then were connected to a volume-controlled ventilator (Servo ventilator 900C^®^, Siemens, Germany). MV was performed in the supine position with a continuous infusion of midazolam (0.06 mg.kg-1.h-1) (Hypnovel^®^, Roche, Switzerland) and cisatracurium besilate (0.3 mg.kg-1.h-1) (Nimbex^®^, GlaxoSmithKline, U.K.). The tidal volume was set at 12 ml/kg with zero end-expiratory pressure in order to submit the lung to a cyclic stretch magnitude likely to drive TLR2 up-regulation as previously described [19]. In addition, the respiratory rate was set at 25/minute and the inspired fraction of oxygen at 0.5. During the MV, the animals were positioned on a heating blanket to maintain body normothermia, and hydration with isotonic serum was provided intravenously to maintain a stable hemodynamic status in the context of MV. An arterial catheter was inserted in most of these animals for blood sampling and blood pressure monitoring. Arterial blood gas was analyzed every 8 hours and the respiratory rate adjusted to keep PaCO_2_ within a 35–45 mmHg range. Plateau pressure was monitored hourly.

The animals were subjected to MV for 24 hours before being given an intrabronchial instillation of 9.5 log10 CFU/mL of *S*. *aureus* diluted in 0.5 mL of saline, as described above. MV controls received 0.5 mL of saline (n = 5). The animals were then kept under MV for 8 or 24 hours before being killed (n = 6 and 11, respectively).

### Material Harvesting

Blood samples were collected from the arterial catheter (at the start of the experiment, after 24 hours of MV and then 1,2,4 and 8 hours after the intrabronchial instillation) and centrifuged at 1,000 g for 15 minutes at 4°C; the supernatant was divided into 0.5 ml aliquots and stored at -80°C for cytokine concentration measurements.

At the end of the experiment, the animals were killed by an intravenous overdose of thiopental (i.e., 100 mg/kg).

In both groups, the animals were exsanguinated by venous puncture. Autopsies were carried out so that the lungs and spleen were aseptically taken. Immediately after exsanguination, the heart and lungs were carefully removed en block via midline sternotomy.

The left lung was lavaged with 2 mL of sterile saline. The broncho-alveolar lavage fluid (BALF) was centrifuged at 400 *g* and 4°C to pellet the cells and the supernatant was stored at -80°C for further cytokine determination.

The lungs were harvested for ribonucleic acid (RNA) extraction. Additional samples were obtained for microscopic examination.

The remaining lung tissue was homogenized for the determination of cytokine concentrations and for bacterial culture.

The spleen was harvested for RNA extraction, and the remaining spleen tissue was homogenized for cytokine concentration measurements and bacterial culture.

### Cytokine Measurements

TNF-alpha and IL-8 concentrations were measured in plasma, whole blood supernatants, lung homogenates and bronchoalveolar lavage fluid (BALF) supernatants using rabbit specific enzyme-linked immunosorbent assay (ELISA) kit (Uscn Life Science Inc., Wuhan, China). The lower limits of the assays were 15.6 picograms/mL for both TNF-alpha and IL-8.

### Quantitative Reverse Transcriptase (RT)-Polymerase Chain Reaction (PCR)

All gene expression analyses were done 8 hours after lung exposure to either saline, Pam3CysSerLys4 (Pam_3_CSK_4_) or *S*. *aureus*, since peak values were achieved within this timeframe in previous studies [19]. RNA was extracted from lung samples using the RNA GenElute kit (Sigma-Aldrich, St Louis, MO, USA). Quantitative PCR was performed using IQ Sybergreen Supermix (Bio-Rad, Hercules, CA, USA). Melting curves were performed to ensure the presence of a single amplicon. The following primers were used: rTlr2, forward 5’-TGT CTG TCA CCG AAC CGA ATC CAC-3’ and reverse 5’-TCA GGT TTT TCA GCG TCA GCA GGG-3’ [23]; rIl-8, forward 5’-AAC CTT CCT GCT GCT TCT GA-3’ and reverse 5’-TCT GCA CCC ACT TTT TCC TTG-3’; TNFα, forward 5’-CAA GCC TCT AGC CCA CGT A-3’ and reverse 5’- GGC AAT GAT CCC AAA GTA G-3’; and rGapdh, forward 5’-ATG TTT GTG ATG GGC GTG AAC C-3’ and reverse 5’-CCC AGC ATC GAA GGT AGA GGA-3’.

The results are expressed as the fold induction using the ΔCt method [24] since the SB animals were always considered as the baseline condition.

### Histological Analysis

A tissue sample of 1 cm^3^ that focused on a macroscopic lesion from the cranial and caudal lobes was fixed in formalin and embedded in paraffin. Five-micrometre sections were obtained and coloured with haematoxylin and eosin. Two pathologists blinded to the group assignment examined all slides. After viewing approximately five fields per sector under low and high power, each section was assigned a numerical histologic score ranging from 0 to 3 as previously described [25] according to the degree of PMNs infiltration, haemorrhage, and oedema in the interstitial and alveolar spaces: 0 (normal), normal-appearing lung; 1 (mild), mild congestion, interstitial oedema, and interstitial PMNs infiltrate with occasional red blood cells and neutrophils in the alveolar spaces; 2 (moderate), moderate congestion and interstitial oedema with neutrophils partially filling the alveolar spaces but without consolidation; 3 (severe), marked congestion and interstitial oedema, with neutrophilic infiltrate nearly filling the alveolar spaces, or with frank lung consolidation. Atelectasis per se was disregarded and not scored as abnormal. For each criterion, when disagreements occurred between the observers, the definite score was defined as the mean of each pathologist’s score.

### Microbiological Evaluation of Pneumonia

Each pulmonary lobe was isolated from the whole lung, homogenized in sterile water. Bacteria were counted in a sample of this crude homogenate by plating 10-fold dilutions on Chapman agar plates (BioMérieux) and incubating the plates for 24 h at 37°C. For each lobe, the bacterial concentration was adjusted to its weight.

The mean bacterial pulmonary concentration was calculated according to each lobar concentration with lobar weight (e.g., mean concentration = ∑ [lobar concentration x lobar weight]/ lobar weights). The spleen of each rabbit was also removed, crushed, and cultured. An *S*. *aureus*-positive spleen culture was considered a marker of bacteraemia.

### Reagents

The α-Human TLR2 monoclonal antibody (mAb) T2.5 (mouse IgG1κ) was kindly provided by Novimune (Plan-les-Ouates, Switzerland), and its relevant isotype control was purchased from Becton Dickinson Biosciences (Franklin Lakes, NJ, U.S.A.).

Synthetic triacyled lipoprotein Pam_3_CSK_4_ was purchased from Invivogen (San Diego, CA, U.S.A.).

### Whole blood assay

Fresh heparinized blood from either SB or MV rabbits (12 mL/kg, ZEEP over a 48 hour-period) (n = 7 and 5, respectively), was obtained by venipuncture and diluted 1:2 with RPMI 1640. Blood was immediately plated at 120 μL/well in a 96-well plate and incubated for 15 minutes at 37°C.

In some experiments, either mAb T2.5 or IgG1κ were diluted with RPMI 1640 (10 μg/mL in 60 μL final volume) and added to rabbit whole blood at room temperature [9]. One hour later 60 μL of Pam_3_CSK_4_ (100 ng/mL) or heat-killed *S*. *aureus* (USA300 strain; 9 log CFU warmed at 90°C for 60’) were added and the blood was then incubated for 24 hours at 37°C. This concentration corresponded to the maximal response observed with the TLR2 agonist in preliminary experiments (data not shown). Cell culture supernatants were finally removed and kept frozen at -80°C until IL-8 and TNF- α concentrations were determined.

### Statistical analysis

All data were expressed as the mean±standard deviation or median [Interquartile range] as specified.

Group size determination was based on previous experience [19].

Data were tested for normal distribution with the D'Agostino-Pearson normality omnibus K2 test or with the Kolmogorov–Smirnov test when n was too small.

Continuous variables between the different study groups (i.e., SB vs. MV animals unless otherwise specified) were compared using parametric one-way analysis of variance for normally distributed data if appropriate. When the difference between groups was significant with the above tests, the group or groups that differed from the others were identified using the Bonferroni multiple comparison test. When the data were not normally distributed, the nonparametric Mann-Whitney U test or the Wilcoxon test was applied if appropriate.

For temporally repeated data (i.e. for serum cytokine concentrations), changes over time were assessed in each group using one-way repeated-measures analysis of variance for normally distributed data. Differences between the times were identified using the Bonferroni multiple comparison test after repeated-measures analysis of variance when significance was reached.

All test were two-tailed. A *p* value lower than 0.05 was considered as statistically significant.

GraphPad Prism software was used (GraphPad Software, San Diego California, USA).

## Results

Main supporting information is provided as an electronic zip file (S1 File).

### Mechanical ventilation worsens lung damage and impairs bacterial lung clearance

We developed an animal model of *S*. *aureus* VAP in lungs previously subjected to MV.

**Fig 2:**
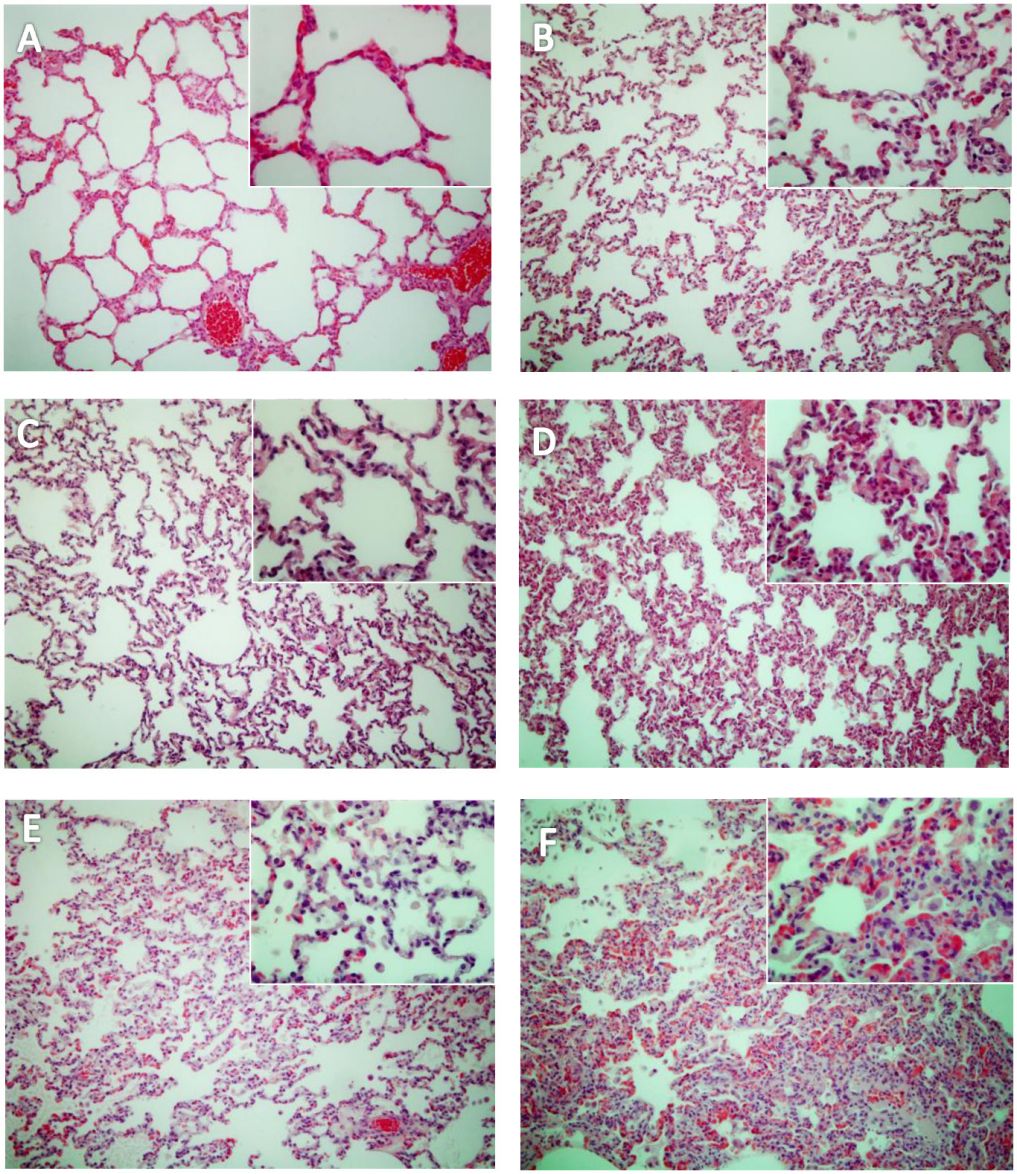
Lung injury main features according to the experimental condition. Six representative light microphotographs of rabbit lungs in various conditions fixed at the same transpulmonary pressure (hematoxylin and eosin x400): (A) spontaneously breathing (SB) healthy rabbit (SB); (B) healthy rabbits submitted to a 32-hours mechanical ventilation (MV); (C) SB rabbits 8 hours after tracheal instillation of 10^9^ CFU of *Staphylococcus aureus*; (D) MV rabbits 8 hours after tracheal instillation of 10^9^ CFU of bacteria; (E) SB rabbits 24 hours after tracheal instillation of 10^9^ CFU of *S*. *aureus*; (F) MV rabbits 24 hours after tracheal instillation of 10^9^ CFU of *S*. *aureus*. We observed interalveolar septa thickened by an interstitial cell infiltrate and several alveoli collapsed mainly in the MV groups (B, D, F). CFU: colony forming unit.

**Fig 3:**
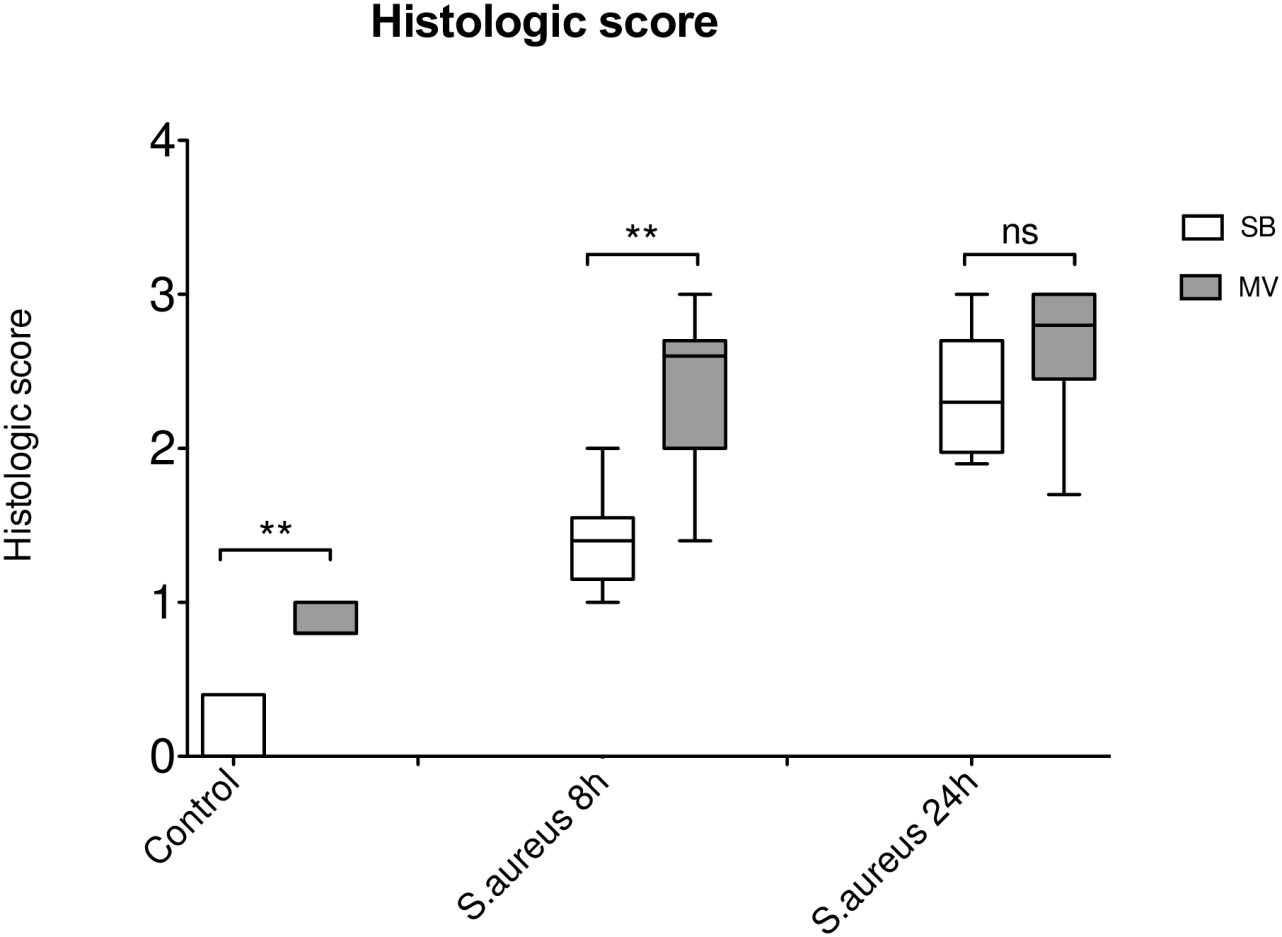
Histologic score according to the experimental condition. Histologic score ranging from 0 to 3, based on the degree of polymorphonuclear infiltration, haemorrhage, and oedema in the interstitial and alveolar spaces, following tracheal instillation of saline (controls) in either spontaneously breathing (SB, n = 5) rabbits or animals subjected to mechanical ventilation (MV, n = 5). Infected animals were challenged with 10^9^ CFU of *Staphylococcus aureus*. After bacterial challenge animals were kept SB or under MV for 8 hours (*n* = 5 and 6, respectively) and 24 hours (n = 8 and 11, respectively). The histologic score was higher under MV. ** denotes *p*<0.05 between SB and MV animals; ns: not significant. CFU: colony forming unit. Controls SB versus (vs) MV p = 0.002; Mann-Whitney U test, two-tailed. *S*. *aureus* pneumonia SB vs MV 8 hours p = 0.011; Mann-Whitney U test, two-tailed. *S*. *aureus* pneumonia SB vs MV 24 hours p = 0.216; Mann-Whitney U test, two-tailed.

As previously reported, 32 hours of MV alone induced lung damage, including inter-alveolar septa thickened by cell infiltrate and alveoli loss of aeration (Fig 2A and 2B). The histological score (Fig 3) was significantly greater in the MV group (0.13±0.20 *versus [vs*.*]* 0.86±0.10, *p* = 0.002). In the setting of pneumonia, 8 hours after tracheal instillation of *S*. *aureus*, we observed increased cellularity, predominantly neutrophils, and fibrin stranding in alveoli, once again predominantly in the MV group (Fig 2C and 2D), resulting in a worse histological score (1.40±0.33 *vs*. 2.40±0.55, *p* = 0.011). Later during the course of infection, (i.e., 24 hours after bacterial challenge), lung injury was still greater in the MV group (Fig 2E and 2F), but the scores did not differ significantly anymore (2.35±0.42 *vs*. 2.67±0.49, *p* = 0.216). Altogether, these findings suggest that lung damage was more severe and earlier in animals subjected to MV and pneumonia than in SB animals with pneumonia.

**Fig 4:**
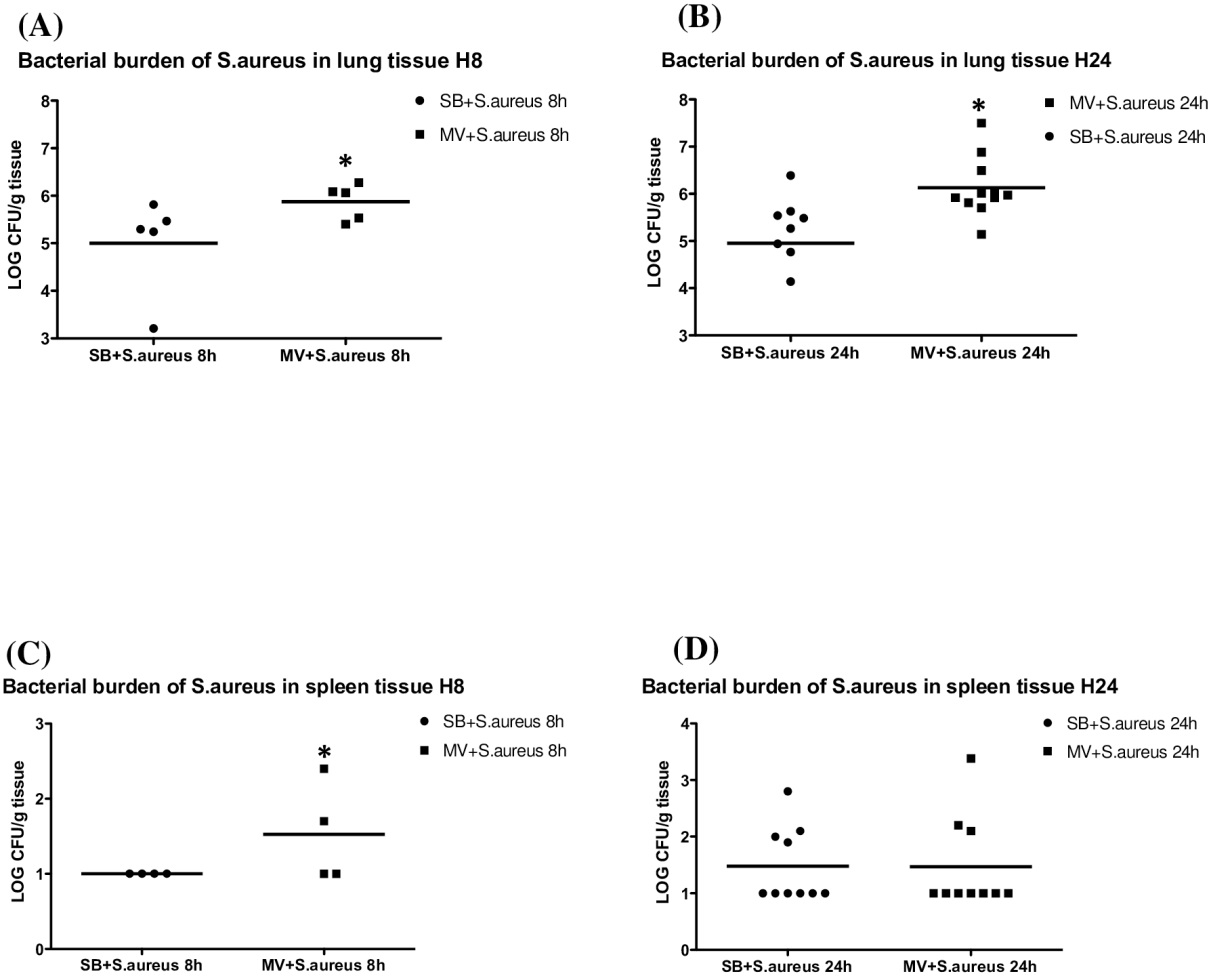
Pulmonary and systemic bacterial burden of *Staphylococcus aureus*. **(4A)** ***S***. ***aureus*** **lung concentrations 8 and 24 hours after bacteria instillation.** Pulmonary bacterial burden according to the experimental group in either spontaneously breathing (SB + *S*. *aureus*) or mechanically ventilated (MV + *S*. *aureus*) animals with *Staphylococcus aureus* pneumonia 8 hours after inoculation (n = 5 and 6, respectively) and 24 hours after inoculation (n = 8 and 11 respectively). The bacterial burden was higher in the MV group both after 8 hours (5.00±1.03 versus [vs.] 5.87±0.38 log_10_CFU/g, *p* = 0.028) and 24 hours of MV (4.96±1.31 vs. 6.13±0.63 log_10_CFU/g, *p* = 0.002). *denotes *p*≤0.05. CFU: colony forming unit. (4B) *S*. *aureus* spleen concentrations 8 and 24 hours after bacteria instillation. Spleen bacterial burden according to the experimental group in either spontaneously breathing (SB + *S*. *aureus*) or mechanically ventilated (MV + *S*. *aureus*) animals with *Staphylococcus aureus* pneumonia (n = 4 and 5, respectively*). Interestingly, MV appears to promote early pulmonary-to-systemic translocation during *S*. *aureus* pneumonia, as indicated by the quantitative spleen cultures, as an indirect marker of bacteraemia, which were positive only in ventilated animals after 8 hours, even though there was no longer any difference after 24 hours. *spleen was lost in 1 animal from each group. Lung *S*. *aureus* concentrations SB versus (vs) MV 8 hours *p* = 0.050; Mann-Whitney U test, two-tailed. Lung *S*. *aureus* concentrations SB vs MV 24 hours *p* = 0.007; Mann-Whitney U test, two-tailed. Spleen *S*. *aureus* concentrations SB vs MV 8 hours *p* = 0.430; Mann-Whitney U test, two-tailed. Spleen *S*. *aureus* concentrations SB vs MV 24 hours *p* = 0.110; Mann-Whitney U test, two-tailed.

The bacterial burden was higher in the MV group both after 8 hours (5.00±1.02 vs. 5.87±0.38 log_10_CFU/g, *p* = 0.050) and 24 hours of MV (4.96±1.31 vs. 6.13±0.63 log_10_CFU/g, *p* = 0.007) (Fig 4A and 4B).

**Fig 5:**
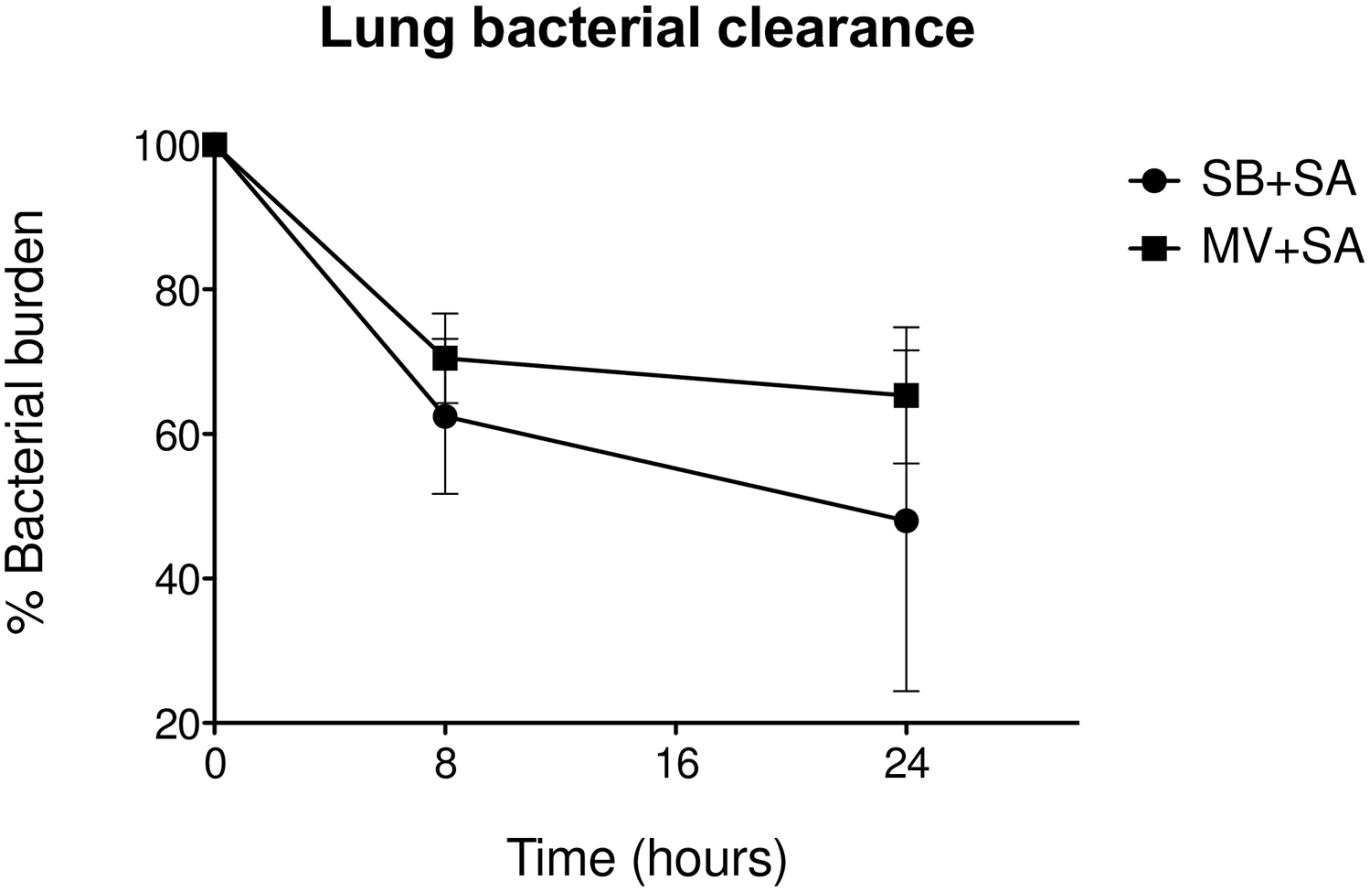
Lung bacterial clearance. Pulmonary bacterial clearance expressed as a percentage of the reduction of log_10_CFU 8 and 24 hours after inoculation with *Staphylococcus aureus* in either spontaneously breathing (SB + *S*. *aureus*; n = 5 and 6, respectively), or mechanically ventilated (MV + *S*. *aureus*; n = 8 and 11, respectively) animals. *denotes *p*≤0.05. CFU: colony forming unit. Pulmonary *S*. *aureus* clearance SB versus MV 24 hours: *p* = 0.045; Mann-Whitney U test, two-tailed.

Similarly, when *S*. *aureus* lung concentrations were expressed as percentages of the inoculum size, bacterial clearance was found to be impaired in MV animals 24 hours after bacterial challenge (65.3 vs. 48.0% of the inoculum in MV and SB respectively, *p* = 0.045) (Fig 5).

In addition, MV probably promoted early pulmonary-to-systemic translocation during *S*. *aureus* pneumonia, as indicated by the quantitative spleen culture results, which reflected the occurrence of bacteraemia (Fig 4C and 4D). Actually, cultures became positive as early as the 8^th^ hour solely in some rabbits in the MV group. Interestingly, *S*. *aureus* spleen concentrations became comparable thereafter (1.48±0.66 *vs*. 1.47±0.82 log_10_CFU/g, *p* = 0.110).

### Mechanical ventilation does not significantly alter the lung inflammatory response to *S*. *aureus*

**Fig 6:**
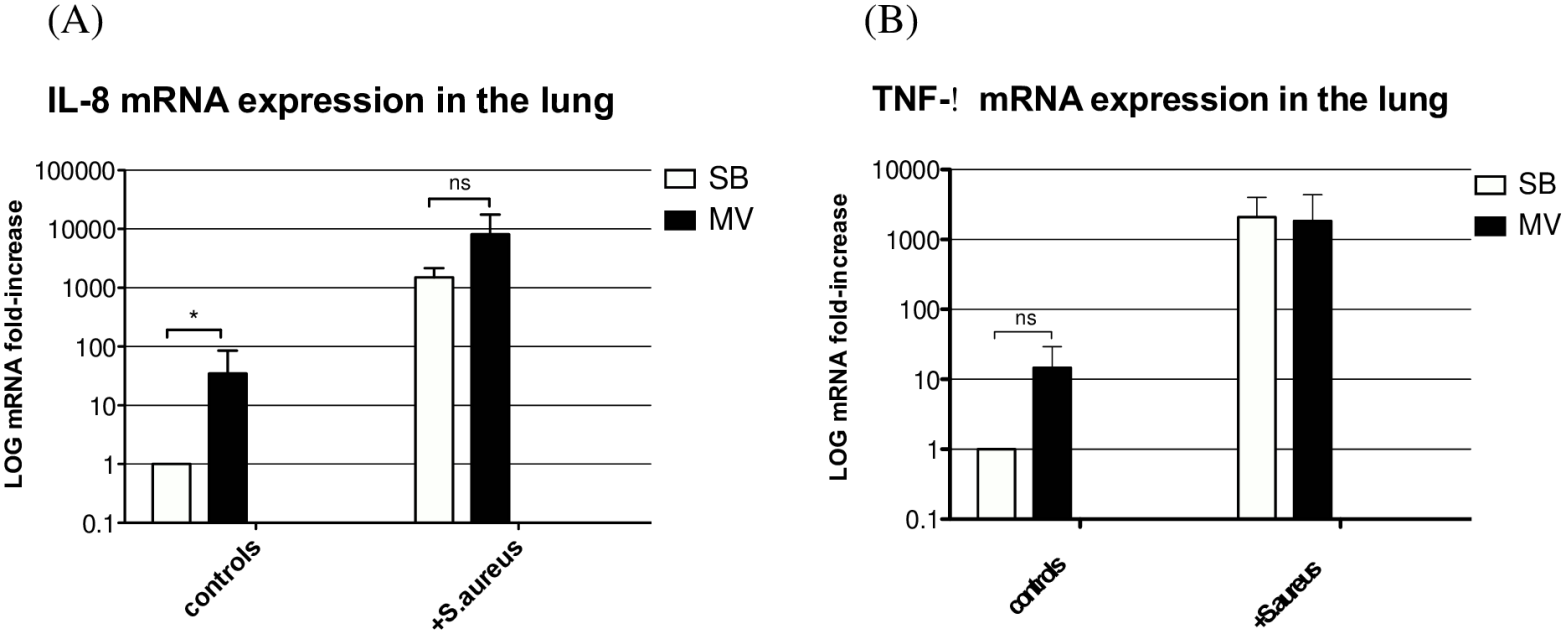
Inflammatory cytokine gene expression in the lung. **(6A) IL-8 gene expression in the lung.** Inflammatory cytokine interleukin (IL)-8 gene expression in lung homogenates 8 hours after tracheal instillation of saline (controls) in either spontaneously breathing (SB, n = 5) rabbits or animals subjected to mechanical ventilation (MV, n = 5), or after challenge with 10^9^ CFU of *Staphylococcus aureus* (+*S*. *aureus*) (n = 5 in each group**). All values are reported as fold increase compared with SB rabbits. Results are expressed as mean±standard deviation. IL-8 gene expression was increased under MV in all groups. *denotes *p*≤0.05; ns: not significant; **PCR amplification failed in 1 MV rabbit. CFU: colony forming unit. Controls SB versus (vs) MV *p* = 0.008, Mann-Whitney U test, two-tailed. *S*. *aureus* SB vs MV p = 0.873, Mann-Whitney U test, two-tailed. (6B) TNF-α gene expression in the lung. Inflammatory cytokine tumor necrosis factor (TNF)-α gene expression in lung homogenates 8 hours after tracheal instillation of saline (controls) in either spontaneously breathing (SB, n = 5) rabbits or animals subjected to mechanical ventilation (MV, n = 5), or after challenge with 10^9^ CFU of *Staphylococcus aureus* (+*S*. *aureus*) (n = 5 in each group). All values are reported as fold increases compared with SB rabbits. Results are expressed as mean±standard deviation TNF-α gene expression was increased under MV only in control animals, *denotes *p*≤0.05; ns: not significant; **Polymerase chain reaction amplification failed in 1 MV rabbit. CFU: colony forming unit. Controls SB versus (vs) MV *p* = 0.076, Mann-Whitney U test, two-tailed. *S*. *aureus* SB vs MV p = 0.825, Mann-Whitney U test, two-tailed.

**Fig 7:**
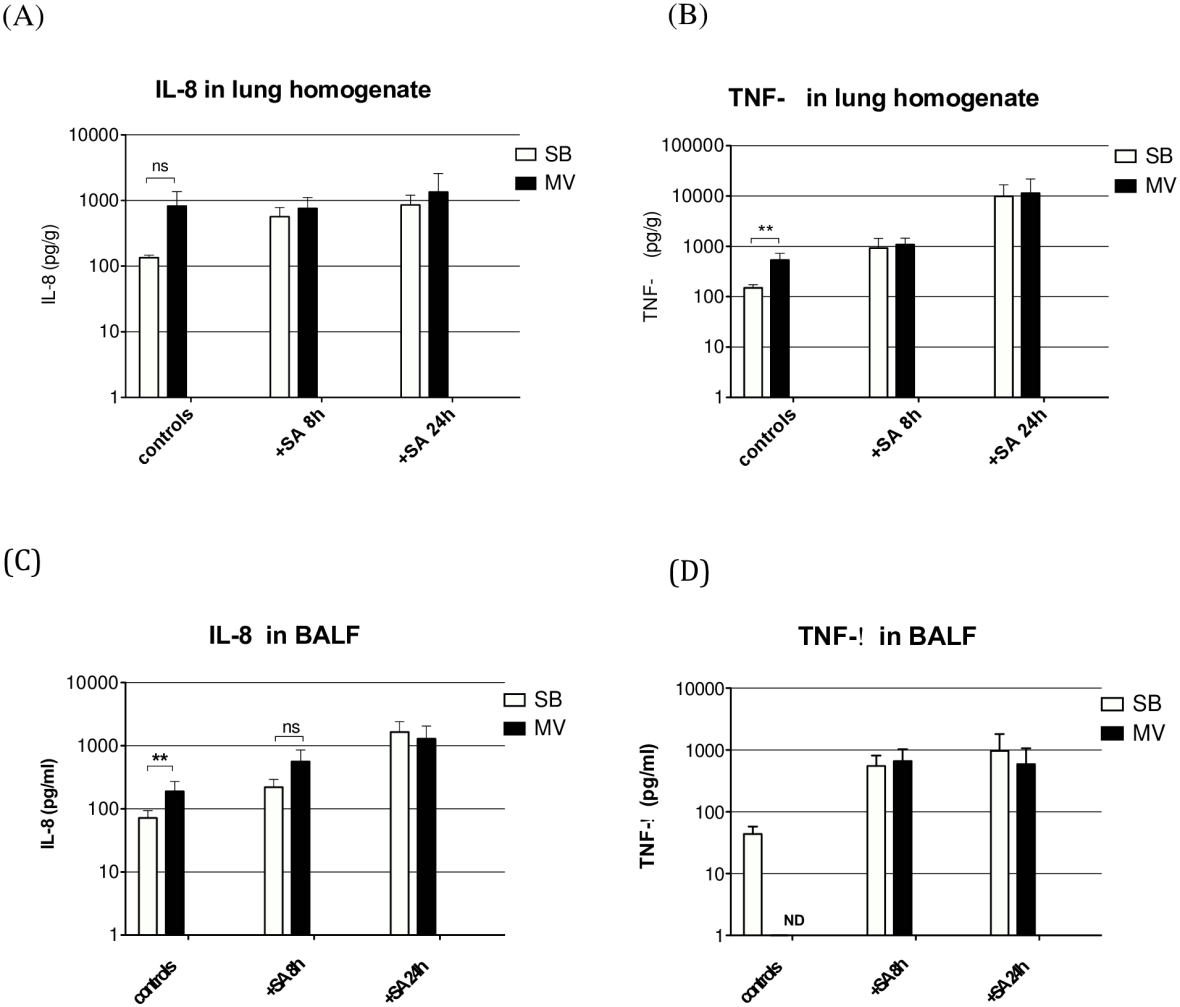
Inflammatory cytokines in the lung. Inflammatory cytokines (interleukin [IL]-8 and tumor necrosis factor [TNF]-α) in lung homogenates and in broncho-alveolar lavage fluid (BALF) 8 to 24 hours after tracheal instillation of saline (controls) in either spontaneously breathing (SB, n = 5) rabbits or animals subjected to mechanical ventilation (MV, n = 5), or 8 hours (n = 5) or 24 hours (n = 10) after challenge with 10^9^ CFU of *Staphylococcus aureus* (+*S*. *aureus* H8 and H24). IL-8 levels were increased in lung homogenates and in BALF under MV in controls, but we did not find any significant difference between SB and MV groups after bacterial challenge. Similarly, TNF-α levels were significantly increased in lung homogenates from animals under MV than in controls, but not after bacterial challenge. ** denotes *p*<0.05; ns: not significant; ND: concentration below the limits of detection of the assay. CFU: colony forming unit. (A) Controls SB versus (vs) MV *p* = 0.333, Mann-Whitney U test, two-tailed. *S*. *aureus* H8 SB vs MV p = 0.397, Mann-Whitney U test, two-tailed. *S*. *aureus* H24 SB vs MV p = 0.395, Mann-Whitney U test, two-tailed. (B) Controls SB vs MV *p* = 0.004, Mann-Whitney U test, two-tailed. *S*. *aureus* H8 SB vs MV p = 0.413, Mann-Whitney U test, two-tailed. *S*. *aureus* H24 SB vs MV p = 0.881, Mann-Whitney U test, two-tailed. (C) Controls SB vs MV *p* = 0.024, Mann-Whitney U test, two-tailed. *S*. *aureus* H8 SB vs MV p = 0.114, Mann-Whitney U test, two-tailed. *S*. *aureus* H24 SB vs MV p = 0.700, Mann-Whitney U test, two-tailed. (D) *S*. *aureus* H8 SB vs MV p = 0.829, Mann-Whitney U test, two-tailed. *S*. *aureus* H24 SB vs MV p = 0.700, Mann-Whitney U test, two-tailed.

In animals subjected to MV alone, lung expression of the inflammatory cytokine IL-8 gene (Fig 6A) was significantly greater than that in their SB counterparts (*p* = 0.031). The corresponding IL-8 protein level within lung homogenates (Fig 7A) and BALF (Fig 7C) tended to be greater in MV animals at the 8^th^ hour. However, the difference was only significant for BALF (71.82±21.80 *vs*. 189.22±83.92, *p* = 0.010) but not for lung homogenate (135.5±11.3 *vs*. 823.6±544.1, *p* = 0.333). In the animals infected with *S*. *aureus*, both IL-8 gene expression and protein release were higher than in non-infected ones (Figs 6A, 7A and 7C). However, we did not find any significant difference between SB and MV groups.

Regarding TNF-α, another inflammatory mediator of paramount importance, lung gene expression was not significantly increased in animals subjected to MV, whether or not they were infected with *S*. *aureus* (Fig 6B). However, the TNF-α protein level within lung homogenates (Fig 7B) was higher in MV than in SB non-infected rabbits (150.5±24.3 *vs*. 533.1±198.8, *p* = 0.004) but there was no difference between the two groups of *S*. *aureus*-infected rabbits at both time points. In contrast, no significant difference was found when BALF concentrations were considered (Fig 7D).

### Mechanical ventilation exacerbates the systemic inflammatory response to *S*. *aureus*

**Fig 8:**
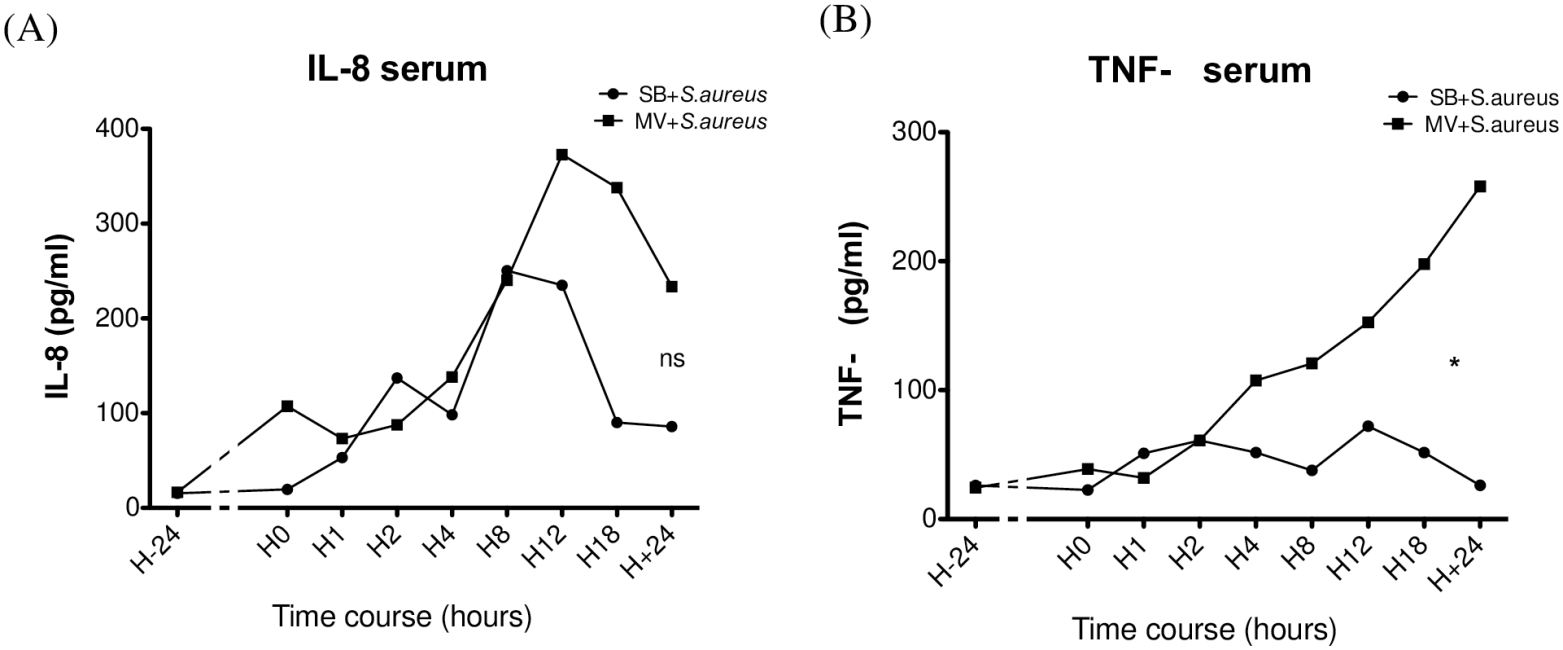
Time course of mean serum inflammatory cytokine concentrations during experiment. Serum concentrations of interleukin (IL)-8 (Fig 8A) and tumor necrosis factor (TNF)-α (Fig 8B) measured by enzyme-linked immune-sorbent assay at baseline, just before intra-tracheal instillation of 10^9^ CFU of *Staphylococcus aureus*, in the spontaneously breathing (SB+*S*.*aureus*) and the mechanically ventilated (MV+*S*.*aureus*) animals, and then 1, 2, 4, 8, 12, 18 and 24 hours after instillation (n = 15). Significant changes over time in each group were assessed by repeated-measures analysis of variance followed by the *post hoc* Bonferroni multiple comparison test when significance was reached. *denotes variations with time significantly different between MV groups versus SB groups; ns: not significant. CFU: colony forming unit. (A) repeated-measures ANOVA mean difference = -69.35 [-141.7 to +2.969] 95% CI; *p* = ns). (B) repeated-measures ANOVA mean difference = -56.11 [-104.7 to -7.547] 95% CI; *p* = 0.010).

Plasma concentrations of IL-8 and TNF-α were measured in animals‘ plasma at baseline and several time-points thereafter (i.e., 1, 2, 4, 8, 12, 18 and 24 h after *S*. *aureus* instillation). Changes over time were assessed by repeated-measures analysis of variance. Time course analysis showed that levels of IL-8 (Fig 8A) at the 8^th^ hour of MV had increased to a greater extent than those measured in SB rabbits, though the difference was not statistically significant. However, a significantly higher TNF-α level was measured in the serum of MV animals than in serum from their SB counterparts (mean difference = -56.11 [-104.7 to -7.547] 95% CI; *p* = 0.010), in spite of the recovery of similar amounts of bacteria from spleen cultures at the last time-point, as described above (Fig 8B).

**Fig 9:**
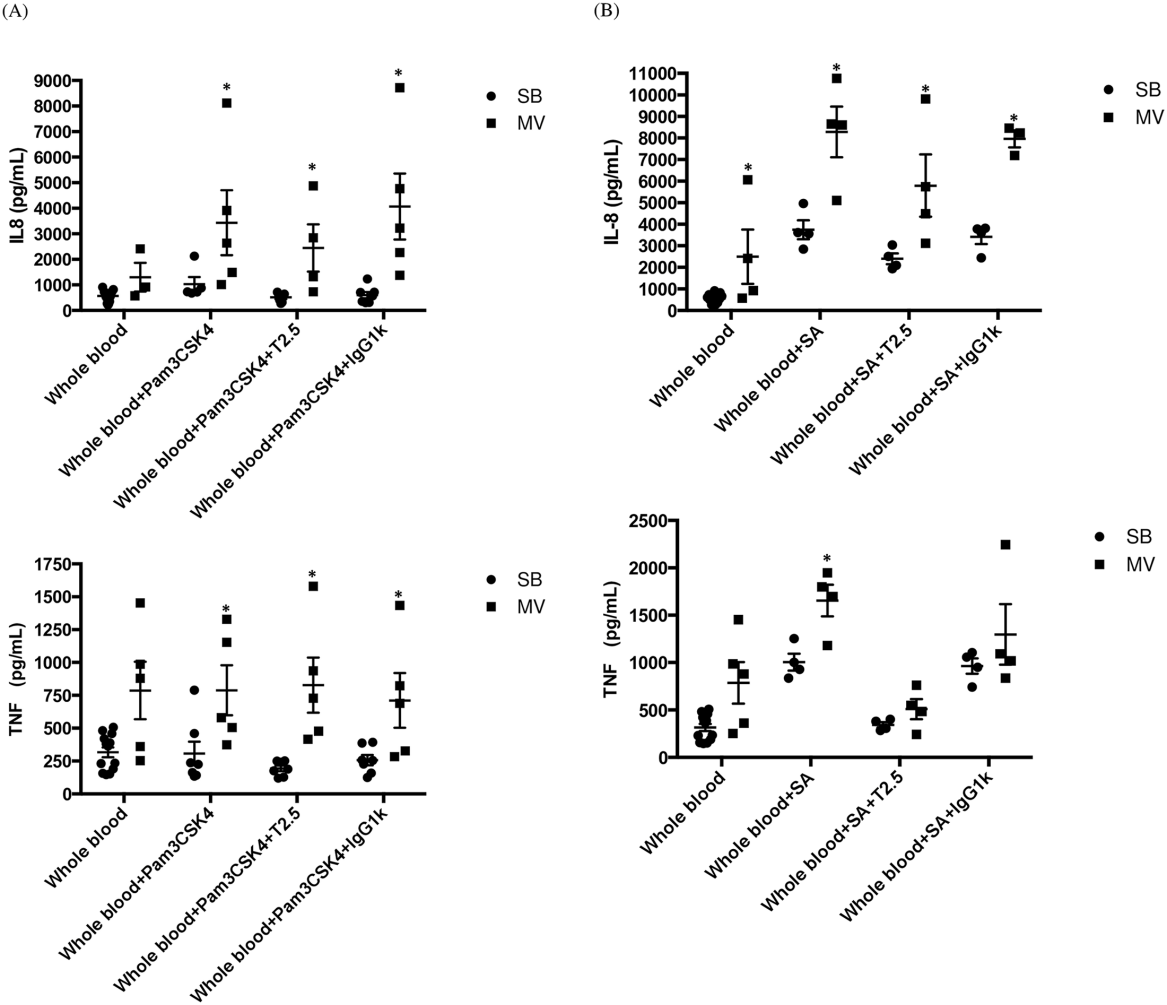
Inflammatory cytokines in whole blood stimulated *ex vivo* by either Pam_3_CSK_4_, or *S*. *aureus*. Interleukin (IL)-8 and tumor necrosis factor (TNF)-α levels in total blood stimulated *ex vivo* by Pam3CysSerLys4 (Pam_3_CSK_4_) (Fig 9A) or *Staphylococcus aureus* (Fig 9B) in either spontaneously breathing (SB, n = 7) rabbits or animals subjected to mild-stretch mechanical ventilation (MV, n = 5), with or without the anti-toll-like receptor (TLR)2 monoclonal antibody (Mab) T2.5. Interleukin-8 levels were not significantly greater in blood obtained from rabbits under MV than in controls but the difference reached statistical significance after stimulation by Pam_3_CSK_4_ and *S*. *aureus* as well. Similarly, TNF-α levels were not significantly higher in blood taken from MV animals than in blood from SB animals. In contrast, Pam_3_CSK_4_ stimulation led to the release of significantly larger amounts of TNF-α in the MV than in the SB group, as did *S*. *aureus* stimulation. Although TLR2 blockade by T2.5 Mab did not abrogate the release of inflammatory mediators by the whole blood obtained from either SB or MV animals after Pam_3_CSK_4_ stimulation, TNF-α but not IL-8 release reached baseline values when Mab was added to media prior to *S*. *aureus* stimulation. * denotes *p*<0.05 between SB and MV animals. (A) IL-8 Controls: SB versus (vs) MV *p* = 0.010, Mann-Whitney U test, two-tailed. Pam_3_CSK_4_ SB vs MV *p* = 0.010, Mann-Whitney U test, two-tailed. Pam_3_CSK_4_ + T2.5 SB vs MV *p* = 0.002, Mann-Whitney U test, two-tailed. Pam_3_CSK_4_ + IgG1k SB vs MV *p* = 0.002, Mann-Whitney U test, two-tailed. TNF-α: Controls SB vs MV *p* = 0.075, Mann-Whitney U test, two-tailed. Pam_3_CSK_4_ SB vs MV *p* = 0.030, Mann-Whitney U test, two-tailed. Pam_3_CSK_4_ + T2.5 SB vs MV *p* = 0.002, Mann-Whitney U test, two-tailed. Pam_3_CSK_4_ + IgG1k SB vs MV *p* = 0.030, Mann-Whitney U test, two-tailed. (B) IL-8: Controls SB vs MV *p* = 0.010, Mann-Whitney U test, two-tailed. *S*. *aureus* SB vs MV p = 0.029, Mann-Whitney U test, two-tailed. *S*. *aureus* + T2.5 SB vs MV p = 0.029, Mann-Whitney U test, two-tailed. *S*. *aureus* + IgG1k SB vs MV p = 0.200, Mann-Whitney U test, two-tailed. TNF-α Controls SB vs MV *p* = 0.075, Mann-Whitney U test, two-tailed. *S*. *aureus* SB vs MV p = 0.057, Mann-Whitney U test, two-tailed. *S*. *aureus* + T2.5 SB vs MV p = 0.343, Mann-Whitney U test, two-tailed. *S*. *aureus* + IgG1k SB vs MV p = 0.657, Mann-Whitney U test, two-tailed.

In another set of experiments, IL-8 and TNF-α were measured in whole blood obtained from either SB or MV rabbits. *Ex vivo* stimulations by the synthetic bacterial lipopeptide Pam_3_CSK_4_, a specific TLR2 agonist, as well as heat-killed *S*. *aureus* were performed in each group in an attempt to mimic *S*. *aureus* cell-wall products release from the lung and bacteraemia, respectively. As expected, in the absence of any microbial stimulation, a mild but not significant elevation of both IL-8 and TNF-α was measured in blood obtained from MV animals when compared to SB ones (Fig 9A and 9B). Whereas Pam_3_CSK_4_ alone failed to elicit the release of inflammatory mediators from the whole blood drawn in SB animals, it mounted a significant response in the MV group. Moreover, our data suggest a synergic effect of the combination of MV with the TLR2 agonist if considering TNF-α release. Similarly, the responsiveness of whole blood from MV animals to heat-killed *S*. *aureus*, in terms of both IL-8 and TNF-α elevation was significantly greater that in blood from SB animals (e.g., TNF-α: 1656±166 vs. 1005±89; *p* = 0.014).

### Mechanical ventilation increases both lung and systemic expression of TLR2

Attempting to explain *in vivo* differences regarding inflammatory responsiveness to *S*. *aureus* between SB and MV rabbits in both the pulmonary and systemic compartments, TLR2 gene expression was evaluated in lung and spleen homogenates, respectively. Spleen was actually considered a surrogate for peripheral blood mononuclear cells [26]. In addition, TLR2 blockade was achieved through media incubation with the T2.5 Mab prior to stimulation during *ex vivo* experiments.

**Fig 10:**
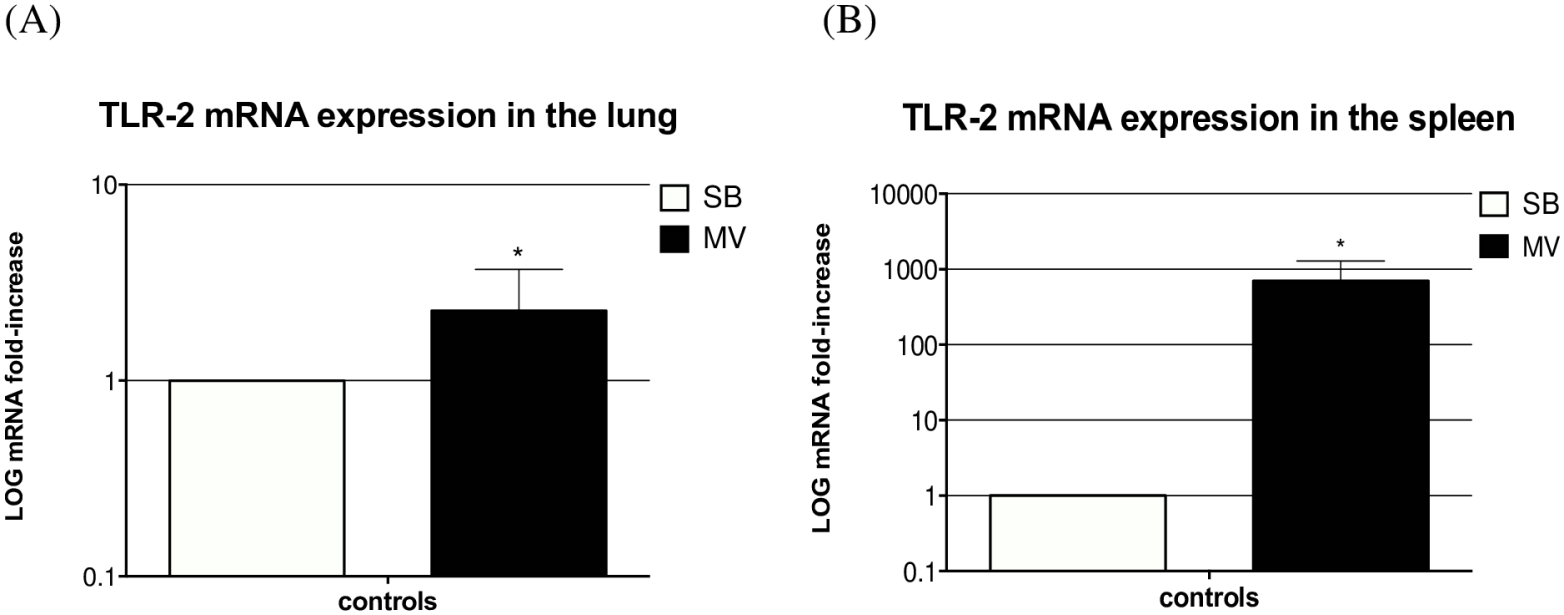
Toll-like receptor 2 gene expression. TLR2 gene expression was measured in both lung (left) and spleen homogenates (right) in either spontaneously breathing rabbits (SB) or animals subjected to mechanical ventilation (MV) (n = 5 in each group). All values are reported as fold increases compared with SB rabbits. Results are expressed as mean±standard deviation. * denotes *p*<0.05 between SB and MV animals. (A) Controls SB versus (vs) MV *p*<0.001, Wilcoxon test, two-tailed. (B) Controls SB vs MV *p*<0.001, Wilcoxon test, two-tailed.

It is worth noting that lung expression of the TLR2 gene was significantly increased under MV (Fig 10A). Similarly, and to a greater extent, MV promoted TLR2 gene expression in spleen homogenates (Fig 10B). However, interestingly, TLR2 *ex vivo* blockade failed to abrogate the excessive release of inflammatory mediators by whole blood obtained from MV animals, excepting TNF-α in response to heat-killed *S*. *aureus* (Fig 9A and 9B). These findings suggest that TLR2 overexpression in MV animals could account, at least in part, for the overwhelming systemic inflammatory response.

## Discussion

In line with our previous data regarding stretch-dependent lung TLR2 overexpression, the aim of this study was to find out to what extent MV could alter the immune and inflammatory response to *S*. *aureus* in a rabbit model of VAP. To this purpose, we challenged the airways with a strong *S*. *aureus* inoculum in animals subjected to a 24 hour-MV prior to infection, and compared them to SB infected controls at both “early” (i.e., 8 hours), or “late” (24 hours) stages of pneumonia.

We report herein the following findings:

- MV impairs bacterial lung clearance and to a lesser extent promotes early pulmonary-to-systemic translocation during *S*. *aureus* pneumonia
- MV worsens lung damage including PMN infiltration, without increasing the local release of inflammatory mediators including IL-8.
- in contrast, systemic inflammation was strongly exacerbated by MV, perhaps reflecting an increased responsiveness to bacterial products within this compartment as shown *ex vivo*.

These results are consistent with our previous findings [19, 21, 22, 27, 28]. However, it is worth noting that some differences exist with respect to the pathogen involved. In addition, the present study provides new insights regarding the impact of MV on bacterial pneumonia.

First, the present data show that the lung’s ability to keep *S*. *aureus* growth in check is impaired by MV. This deleterious effect of MV becomes obvious at the early stage of infection (i.e., at the 8^th^ hour). Interestingly, these findings are in accordance with previous reports from our group concerning other pathogens (e.g., *Enterobacter* sp. and *Streptococcus pneumoniae*). Moreover, to the best of our knowledge, this is the first study to demonstrate such major deleterious effects of MV in a MRSA VAP model, thereby illustrating the peculiar severity of these infections. Dhanireddy *et al*. failed to demonstrate any significant impact of lung stretch on *S*. *aureus* clearance in mice with pneumonia subjected to 12 hours of MV [14]. Our model, however, is more relevant to the clinical scenario of VAP since animals were subjected to 24 hours of MV prior to bacterial challenge. In addition, it has been shown that rabbit TLRs are more closely related to human TLRs than to mouse TLRs [23]. In addition, if translated into the clinical area, our findings might reflect the reported quite high treatment failure rate despite the administration of highly active drugs [29]. Similarly, we showed that MV could promote pulmonary-to-systemic bacteria translocation but only at the earliest stage of infection. Actually, similar spleen bacterial concentrations were found in SB and MV rabbits after 24 hours despite significantly greater lung concentrations in MV animals. This result was not expected since cumulative experimental evidence supports the hypothesis that MV promotes bacterial spill over from the lung [13, 21, 30]. Moreover, clinical data showed that *S*. *aureus* VAP was more likely to be associated with bacteraemia than was pneumonia caused by other pathogens [31, 32]. Our findings are, however, in line with those obtained previously in mice [14]. In addition, the propensity of bacteria to reach the bloodstream depends on the host immune response as well as virulence factors [33]. Indeed, in this study we used a PVL-deleted USA300 strain. Interestingly, our group has recently shown in rabbits that bacteraemia was achieved in every animal if pneumonia was caused by the original USA300 strain (i.e., PVL+), thus emphasizing the role of the toxin [20]. Of note, we chose the PVL- strain because of the expected high mortality rate associated with toxin production as previously reported, which would have not provide us the opportunity to evaluate the effect of such a long-term MV.

Regarding lung damage, the present findings confirmed previous ones, since *S*. *aureus* caused significantly greater injury in animals subjected to MV than in their SB counterparts. However, although this difference was obvious at the early stage of pneumonia (i.e., 8 hours after bacterial challenge), lung disease severity was similar at the 24^th^ hour, suggesting that lung stretch might have hastened the spread of tissue injury, especially PMN infiltration. This phenomenon has already been described in animal models of VILI, which showed that the more adverse the MV settings, the earlier the VILI features became obvious [34]. However, these findings do not clearly support the hypothesis that MV primes the lung before bacterial challenge occurs within the airways, thus driving lung overreaction, in accordance with the “two-hit” paradigm [35]. Actually, the experimental design does not allow us to determine whether the reported effects of MV were related to MV “pre-exposure” and/or to the MV period following bacterial challenge. Nevertheless, we have hypothesized and demonstrated previously in both human cells and rabbit lung that stretch-dependent TLR2 overexpression in the lung could account for lung priming [19]. Stretch-dependent TLRs 2 and 4 have also been demonstrated in other animal models of VILI [36, 37]. We should, however, admit that the data provided by the present study using live bacteria instead of one specific TLR2 agonist were not so consistent. Although pulmonary *S*. *aureus* concentrations were quite similar at the early time-point (i.e., 8^th^ hour), it is worth noting that the release of neither IL-8 nor TNF-α in the lung was significantly increased by the combination of MV and infection, despite the fact that greater amounts of bacteria were simultaneously recovered from the lung. Several explanations could be considered. Firstly, some authors have discussed the relevance of biotrauma in animal models of VILI, arguing that the mechanical insult *per se* could account for tissue injury [38]. Secondly, lung reactivity to *S*. *aureus* might depend on the bacterial strain, as recently reported [39]. We cannot therefore exclude the possibility that lipoprotein expression within the *S*. *aureus* wall changes not only with time but also according to the underlying environmental conditions (e.g., according to lung stretch). Moreover, it has been shown that the activation profile of circulating neutrophils in the pulmonary and extra-pulmonary blood flow varies according to lung conditions [40]. It is therefore possible that the excess lung damage was subsequent to primed neutrophil retention rather than to TLR2 overexpression, as described in patients with acute respiratory distress syndrome (ARDS). Finally, since IL-8 lung release was not increased by MV, other PMN chemoattractant mediators might be involved, acting as pro-inflammatory signals other than the usually assessed chemokines and cytokines. Indeed, the involvement of so-called alarmins in the onset of VILI has recently been raised and needs further investigation [41].

Our study also addressed the issue of systemic inflammatory response in the setting of *S*. *aureus* VAP. As mentioned above, we failed to demonstrate clearly that MV associated with bacterial challenge increased lung inflammation, as reflected by pulmonary gene expression or cytokine concentrations. However, and in contrast, we observed a rising systemic inflammatory response (i.e., plasma levels of IL-8 and TNF-α), as early as the 8^th^ hour only in animals with pneumonia subjected to MV. It is worth noting that the amount of circulating live bacteria, according to spleen cultures, was mild over this period, regardless of ventilation. One cannot exclude, however, the possibility that lung stretch promoted the translocation of killed bacteria or bacterial products (e.g., cell wall components) from the lung, as shown with LPS, since lung damage was found to be more severe in the MV group [13]. Inflammatory mediator spill over from the lung should not be ruled out but seems unlikely since similar concentrations were measured within the tissue.

In order to investigate this issue, a second set of experiments was carried out, with special emphasis on the role of TLR2. Our results suggest that the systemic inflammatory response observed in this model of VAP depended, at least in part, on a TLR2 pathway in peripheral white blood cells. Actually, we showed that MV alone led to TLR2 gene overexpression in the spleen. Indeed, the results of *ex vivo* experiments suggest that the whole blood obtained from rabbits subjected to MV overreacted to one TLR2 agonist and to heat-killed *S*. *aureus* as well. However, blocking TLR2 with a specific monoclonal antibody did not completely abrogate the release of IL-8 in this setting, in contrast with TNF-α which concentrations dramatically fell until reaching levels near from baseline values.

Altogether, these data suggest that the activation of pathways different from TLRs could also be involved, accounting thereby for the overwhelming and protracted systemic inflammatory response we measured in animals with both MV and pneumonia. Indeed, we could hypothesize that in the context of *S*. *aureus* pneumonia, other pro-inflammatory signals are released in much larger amounts into the blood compartment of animals subjected to MV than in SB ones. These could be alarmins, released by the injured lung and likely to activate the host response through the inflammasome. Interestingly, as previously described, each pathway could drive the production of different inflammatory mediators. Indeed, IL-8 and TNF-α behaved differently in our *ex vivo* experiments when TLR2 was blocked, suggesting that IL-8 production did not depend on the TLR2 pathway alone.

In addition, a recently published study showed that if “primed” PMNs crossed the alveolo-capillary barrier, they were “deprimed” thereafter as leaving the pulmonary circulation, provided the lungs were healthy [40]. In contrast, the authors observed that in ARDS patients, this depriming process was impaired, thus leading in turn to the circulation of activated inflammatory cells toward extra-pulmonary organs. Of course, further experiments are necessary to clarify these issues. It is worth noting for instance that the PMN activation profile was not assessed in the present study.

Nevertheless, our data suggest that in addition to the lung, the systemic compartment was likely to overreact to microbial challenge, thus accounting, at least in part, for the greater degree of inflammation measured in MV animals.

Several limitations of our study should be mentioned. First, the MV without PEEP we used could be considered as non-clinically relevant, since it is likely to promote cyclic opening and closing of lung units. However, it has recently been reported that such settings were still used, especially in the operating room [42]. In addition, it is known that lung injury is heterogeneous in ARDS patients. As a result, poorly aerated areas of the lung usually coexist with overstretched ones, even if “low-V_T_” (i.e., 6 mL per ideal body weight) is applied, as shown in human studies [43]. Although cautiously, one could assume that such parts of the lung may react as described in the present study if bacterial challenge with *S*. *aureus* occurred in patients subjected to MV. However, adding a group of animals subjected to some more protective MV settings would be of interest. Second, TLR2 assessment was limited to gene expression. We cannot therefore draw any strong conclusions regarding this point since the corresponding protein was not measured directly within the lung or the spleen. Moreover, it is uncertain whether epithelial cells, macrophages or PMNs expressed TLR2 the most. We should also acknowledge that by using SB animals as controls, lung stretch was not the only factor evaluated. Actually, one cannot exclude the possibility that other mechanisms, such as impaired airway drainage subsequent to tracheal intubation, prolonged supine position and general anesthesia as well, could account, at least in part, for the lower bacterial clearance rate we report here. However, we have previously published data showing that when mild-stretch MV was compared with low-stretch MV in a Gram-negative bacteria VAP model, the latter strategy was likely to favor the host regarding this point [28]. In addition, we used one bacterial inoculum which size could be considered as greater than usually encountered in the clinical setting. Finally, the fact that animals were not given antibiotics made questionable any translation to the clinical area. Further experimental studies are therefore needed.

Even although the first experimental evidence of VILI was published 40 years ago [44], the so-called lung-protective MV settings became the recommended standard of care only in the early 2000s thanks to the ARDS network publication [45]. This strategy thus remains restricted to patients with ARDS. However, most preclinical studies showed that MV using low tidal volumes was likely to dampen the injurious effects of MV in animals with previously healthy lungs [46, 47]. Today, there is growing evidence that the use of lower tidal volumes also benefits patients who do not have obvious lung injury. In a recent meta-analysis, some authors showed a significantly lower incidence of pulmonary infection with the use of lung-protective ventilation with lower tidal volumes [48]. Others showed a reduction in major pulmonary complications requiring MV (i.e. pneumonia) with lung-protective MV in patients who underwent major abdominal surgery [49]. Finally, despite the growing knowledge in this field, up to 16% of patients continue to receive non-protective ventilation in the operating room [42]. Altogether, these data strongly suggest that MV-related lung-stretch could be harmful, even if applied to apparently healthy tissue, maybe because of an increased risk of severe pneumonia if the airways are challenged with bacteria. Our findings thus send a word of warning to physicians who deal with mechanically ventilated patients, especially if these patients develop bacterial pneumonia.

## Conclusions

In a rabbit model of *S*. *aureus* VAP, we showed that mild-stretch MV was likely to impair lung bacterial clearance, hasten tissue injury, and promote a systemic inflammatory response. TLR2 overexpression in both pulmonary and peripheral blood may play a role, but other factors likely to drive such overwhelming inflammation should be sought.

## Supporting Information

S1 File Main raw data and statistical analysis reports. (ZIP)
